# Engineering the Future of Bladder Repair: Can Biocompatible 3D-Printed Scaffolds Serve as a Novel Alternative to Intestinal Segments for the Treatment of Bladder Exstrophy?

**DOI:** 10.1155/aiu/9437696

**Published:** 2025-09-10

**Authors:** M. Forooghi, A. Askari, M. Haghdel, A. G. Haghighi, M. H. Anbardar, A. H. Hassani, H. Foroutan, A. S. Aloudal, Sh. Yousufzai

**Affiliations:** ^1^Department of Pediatric Surgery, Namazi Hospital, Shiraz University of Medical Sciences, Shiraz, Iran; ^2^Student Research Committee, Shiraz University of Medical Sciences, Shiraz, Iran; ^3^Tissue Engineering & Applied Cell Sciences Department, School of Advanced Medical Sciences & Technologies, Shiraz University of Medical Sciences, Shiraz, Iran; ^4^Thoracic Surgery Department, Namazi Hospital, Shiraz University of Medical Sciences, Shiraz, Iran; ^5^Department of Pathology, Shiraz Medical School, Shiraz University of Medical Sciences, Shiraz, Iran; ^6^Urology Department, Namazi Hospital, Shiraz University of Medical Sciences, Shiraz, Iran

**Keywords:** 3D-printed scaffold, bladder exstrophy, bladder reconstruction, polylactic acid (PLA), polyvinyl alcohol (PVA), thermoplastic polyurethane (TPU)

## Abstract

**Background:** Bladder reconstruction traditionally involves intestinal segments, which, despite their effectiveness, carry significant risks such as metabolic disturbances and infection. Safer, synthetic alternatives are needed. We evaluated a novel 3D-printed multilayered bladder scaffold combining polylactic acid (PLA), thermoplastic polyurethane (TPU), and polyvinyl alcohol (PVA) in a rabbit model.

**Methods:** Anatomically tailored scaffolds were designed using computer-aided design (CAD) and fabricated under good manufacturing practice (GMP) conditions. Mechanical integrity was assessed after 60 days of incubation in simulated bladder media, including measurements of modulus of elasticity, tensile strength, elongation, and shape recovery. Acid/alkaline resistance was tested for chemical stability. For in vivo analysis, four rabbits underwent bladder augmentation with a 1 × 1 cm scaffold-augmented defect. Postoperative outcomes were monitored for 60 days, followed by histopathological evaluation.

**Results:** After incubation, the scaffolds retained mechanical strength (modulus: 1.2 ± 0.3 GPa; tensile strength: 18.5 ± 2.1 MPa) with minimal elongation reduction (25% vs. 28% unused). Chemical testing confirmed structural stability and full shape recovery. In vivo, all rabbits survived without urinary leakage. Mild intra-abdominal adhesions and universal cystolithiasis were noted. Histology showed complete urothelial reepithelialization and mild-to-moderate submucosal fibrosis with chronic inflammation but no necrosis or acute inflammation. Compared to biological scaffolds, the synthetic construct showed reduced mortality and comparable inflammation, though with increased stone formation.

**Conclusion:** This 3D-printed scaffold demonstrates promising biocompatibility, mechanical durability, and integration in bladder repair. While early results are encouraging, further studies with larger sample sizes and longer follow-up are needed to address limitations such as cystolithiasis risk.

## 1. Introduction

Bladder reconstruction is a crucial intervention in the management of complex lower urinary tract disorders, particularly in cases where conservative therapy has proven ineffective. The primary clinical indications for this procedure include neurogenic bladder dysfunction, the bladder exstrophy-epispadias complex, and complications arising from treatments for pelvic malignancies, such as prostate cancer [1–5].

Neurogenic lower urinary tract dysfunction (NLUTD), which can result from spinal cord injuries or congenital conditions like spina bifida, often necessitates surgical intervention due to persistent incontinence, difficulties with catheterization, and the risk of upper urinary tract damage [1, 2]. Similarly, bladder exstrophy-epispadias, a congenital malformation, along with postoncologic reconstructive needs, frequently requires surgical reconstruction to restore urinary function and enhance the quality of life [3–5].

Enterocystoplasty, which utilizes intestinal segments for bladder augmentation, remains the gold standard for patients with refractory NLUTD, complex exstrophy, or postcancer reconstructive needs [6–9]. This technique significantly increases bladder volume and compliance, improves renal function by alleviating high intravesical pressures, and enhances continence as well as patient-reported quality of life [7–9]. However, it is associated with considerable morbidity. Complications may include metabolic disturbances such as hyperchloremic acidosis, vitamin B12 deficiency, and osteoporosis [10], as well as bowel-related symptoms including diarrhea and fecal urgency [11].

Patients are also at risk for bladder stone formation [12], spontaneous perforation [12], infections, postoperative ileus, and abscess formation, particularly when colonic segments are employed [12, 13]. Consequently, preoperative counseling and lifelong follow-up are emphasized in contemporary guidelines [2]. There is an increasing recognition of the need for safer, functionally robust, and less morbid alternatives to bowel-based augmentation.

Alternative surgical approaches, including gastrocystoplasty [14, 15], ureterocystoplasty [16], and seromuscular enterocystoplasty [17], have been explored; however, they are constrained by technical complexity, the availability of suitable tissue, and insufficient bladder capacity. Tissue engineering strategies, designed to address these challenges, are still under investigation due to ongoing obstacles such as poor mechanical performance, inadequate vascularization, immune responses, and limited innervation [18–27].

Synthetic scaffolds present several advantages over biological matrices, such as bladder acellular matrix (BAM) and xenografts. Materials like polylactic-co-glycolic acid (PLGA) and silk fibroin exhibit minimal immunogenicity and superior mechanical strength [28–31]. In contrast, biological scaffolds frequently encounter immune-related complications, fibrosis, shrinkage, and mechanical failure [28–33]. Research indicates that synthetic materials offer more consistent long-term support for tissue regeneration and maintain mechanical integrity more effectively than their biological counterparts [30, 32, 33].

Among emerging technologies, 3D bioprinting facilitates the fabrication of anatomically precise scaffolds with customized mechanical and biological properties. By enabling the controlled spatial deposition of biomaterials, cells, and bioactive factors, 3D bioprinting enhances scaffold biocompatibility and cellular behavior [34–36]. Optimized scaffold porosity and geometry have been shown to support high cell viability and proliferation [37]. Hybrid 3D bioprinting approaches, such as those that combine PLGA microspheres with hydrogels, significantly improve mechanical resilience, which is crucial for the dynamic environment of the bladder [38]. Constructs created through these methods demonstrate over a 100-fold mechanical enhancement compared to traditional hydrogels, exhibiting sufficient elasticity and durability to mimic native bladder tissue [38, 39].

Recent research underscores the significance of material selection in scaffold design. Polylactic acid (PLA) provides biodegradability, biocompatibility, and the mechanical strength required for bladder support [40–42]. Thermoplastic polyurethane (TPU) introduces elasticity and flexibility, which are essential for accommodating bladder filling and voiding cycles [43–45]. Additionally, polyvinyl alcohol (PVA) enhances hydrophilicity, promotes cell adhesion, and ensures structural stability under physiological conditions [46, 47]. By integrating PLA, TPU, and PVA, it is possible to fabricate scaffolds with optimal properties for bladder tissue integration and regeneration [23, 42–44, 47, 48].

This study addresses a significant gap in the literature by evaluating a novel, multilayered 3D-printed bladder scaffold composed of PLA, TPU, PVA—materials that have not been previously validated in this composite form for bladder augmentation. Unlike prior studies that have predominantly focused on biological scaffolds, this research integrates computer-aided design (CAD)–guided 3D printing to achieve anatomical accuracy and mechanical robustness.

The study aims to (1) design a multilayered scaffold with biomechanically appropriate components using FDA-approved materials; (2) assess mechanical properties before and after in vivo implantation through uniaxial tensile testing; (3) evaluate surgical feasibility and scaffold integration in a rabbit bladder augmentation model over a 60-day period; and (4) investigate histological outcomes, including reepithelialization, fibrosis, inflammation, and cystolithiasis.

## 2. Materials and Methods

### 2.1. Animal Models and Surgical Procedures

This study utilized adult white rabbits sourced from the Research Center of our medical institution under reference ID “IR.SUMS.AEC.1401.022.” The animals were housed individually in standardized laboratory cages measuring 40 × 50 cm, under controlled conditions that included a 12-hour light/dark cycle and an ambient temperature of 25°C. All rabbits had unrestricted access to standard laboratory chow and water, and designated resting areas were provided to ensure environmental enrichment and minimize stress throughout the study period.

Anesthesia was induced through the intravenous administration of 10% ketamine hydrochloride (20 mg/kg) and 2% xylazine hydrochloride (4 mg/kg), both obtained from ALFASAN, Netherlands, via the marginal ear vein. Anesthesia was maintained with a continuous intravenous infusion of propofol at a rate of 0.4 mg/kg/h, supplemented with 0.5% isoflurane delivered via a facemask to ensure a consistent anesthetic depth throughout the surgical procedure.

**Figure 1:**
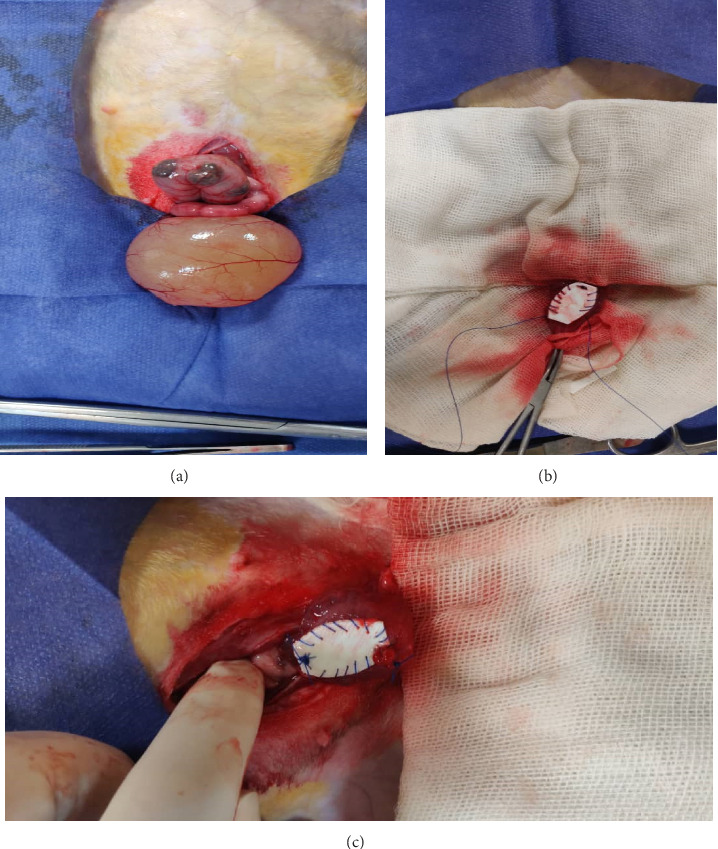
(a–c) Surgical repair of bladder exstrophy in a rabbit model using a 3D-printed biodegradable scaffold. (a) Prolapsed bladder with exposed mucosa and edematous urothelium preimplantation. (b) Placement of a tailored 3D-printed scaffold onto the bladder defect, secured with interrupted sutures. (c) Final suturing step, showing full integration of the scaffold within the native bladder wall margins.

Under sterile conditions, a midline lower abdominal laparotomy was performed to expose the bladder. A standardized 1 × 1 cm full-thickness defect was created on the anterior bladder wall using sharp dissection. This defect was promptly repaired with an experimental multilayered synthetic scaffold, which was sutured into place using absorbable polyglactin 910 (Vicryl) sutures to ensure watertight closure and integration with the native bladder tissue (Figures 1(a), 1(b), and 1(c)). Care was taken to avoid injury to surrounding anatomical structures and to preserve the overall integrity of the bladder.

### 2.2. Postoperative Monitoring and Animal Welfare

Postoperative monitoring was conducted daily and included weight measurement, activity and food intake scoring (on a standardized 1–4 scale), and gross assessment of urinary function. Predefined humane endpoints, including > 20% weight loss or severe distress, were established; however, no animals met these exclusion criteria during the study period.

### 2.3. Scaffold Processing and Sterilization

**Figure 2:**
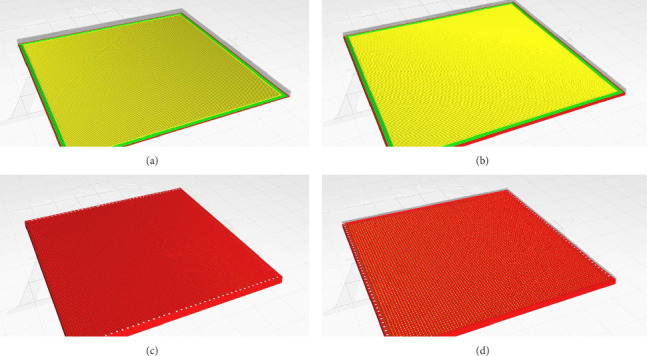
(a–d) A three-dimensional computer-aided design (CAD) of a multilayer bladder scaffold, which comprises the following components: (a) the bottom layer, (b) the mid-layer, (c) the overall structure, and (d) the top layer.

Following fabrication (Figures 2(a), 2(b), 2(c), and 2(d)), scaffolds underwent rigorous postprocessing: initial ethanol/PBS washing to remove residual printing materials, followed by low-temperature ethylene oxide (EtO) sterilization (37°C for 12 h) to preserve the structural integrity of the heat-sensitive PLA and TPU components. Sterility and endotoxin levels were validated via ISO 11737-1 testing and Limulus amebocyte lysate (LAL) assays, respectively, confirming biocompatibility with endotoxin levels < 0.25 EU/mL.

### 2.4. Surgical Implantation Technique

Scaffolds were surgically secured to the bladder using interrupted 5-0 polyglactin (Vicryl) sutures, with 12–14 sutures per implant spaced at 1 mm intervals. Full-thickness suturing through both the scaffold and bladder wall ensured mechanical stability, and intraoperative saline leak testing confirmed anastomotic integrity prior to closure.

### 2.5. Scaffold Design Parameters

The multilayer scaffold was engineered with a total thickness of 1.2 mm, comprising distinct layers of PLA (0.5 mm, structural support), TPU (0.5 mm, elastic compliance), and PVA (0.2 mm, hydrophilic interface). A hexagonal pore architecture (150–200 μm diameter) was implemented to facilitate cellular infiltration while maintaining mechanical stability during tissue remodeling.

### 2.6. Histopathological Analysis

Quantitative histological assessment was performed by two blinded pathologists using standardized scoring systems: inflammation was graded from 0 (absent) to 3 (severe) based on leukocyte infiltration density; epithelial maturation was scored from 1 (discontinuous) to 4 (stratified); and fibrosis was quantified as the percentage of collagen deposition area per high-power field.

### 2.7. Urinary Tract Sterility Assessment

Although microbial cultures were not routinely performed, the absence of clinical infection signs, lack of acute inflammation on histopathology (e.g., neutrophilic infiltrates), and micro-CT analysis of stone microstructure (homogeneous mineral composition without gas pockets) collectively supported the conclusion of sterile lithiasis in this model.

Postoperative care involved close monitoring for signs of pain, infection, or surgical complications. All animals received analgesics and prophylactic antibiotics in accordance with institutional animal care protocols. After recovering from anesthesia, the rabbits were returned to their housing and observed for 60 days, with endpoint evaluations including gross pathology and histopathology.

## 3. Results

After incubation, the scaffolds retained mechanical strength (modulus: 1.2 ± 0.3 GPa; tensile strength: 18.5 ± 2.1 MPa) with minimal elongation reduction (25% vs. 28% unused). Chemical testing confirmed structural stability and full shape recovery. In vivo, all rabbits survived without urinary leakage. Mild intra-abdominal adhesions and universal cystolithiasis were noted. Histology showed complete urothelial reepithelialization and mild-to-moderate submucosal fibrosis with chronic inflammation but no necrosis or acute inflammation. Compared to biological scaffolds, the synthetic construct showed reduced mortality and comparable inflammation, though with increased stone formation.

### 3.1. Scaffold Integration and Degradation

**Figure 3:**
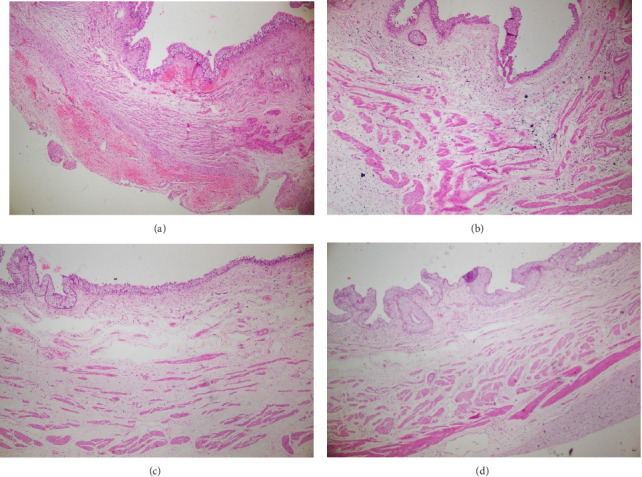
(a–d) Histopathological examination of scaffold implantation sites showed complete reepithelialization with a continuous urothelial lining and mild-to-moderate submucosal fibrosis. Chronic inflammation, evidenced by lymphocytes, plasma cells, and occasional macrophages, was present without signs of acute inflammation or necrosis. H&E staining (40×) highlighted organized cellular architecture and collagen deposition, indicating favorable scaffold integration and biocompatibility.

The scaffold demonstrated progressive tissue integration over the 60-day study period, with mechanical testing data (Table 1) and histopathological evaluation (Figure 3) indicating partial degradation while preserving structural integrity. Notably, tensile strength decreased by 12% (18.5 ± 0.8 MPa to 16.3 ± 0.6 MPa) and elastic modulus by 11% (1.2 ± 0.3 GPa to 1.07 ± 0.2 GPa), consistent with controlled resorption kinetics. Histomorphometry analysis revealed complete epithelial coverage with a mean thickness of 45.2 ± 8.7 μm, approximating native urothelial dimensions, alongside moderate inflammatory responses (mean score 1.25 ± 0.5 on a 0–3 scale). The presence of α-smooth muscle actin-positive (α-SMA+) cells in 75% of specimens (*n* = 4) further suggested early smooth muscle regeneration within the scaffold matrix.

Also, the 3D-printed scaffold featured a multilayer design consisting of a 0.5 mm PLA base layer, a 0.5 mm TPU intermediate layer, and a 0.2 mm PVA top layer, incorporating a hexagonal honeycomb pore geometry with an average pore size of 150–200 μm (Table 2). Histomorphometric analysis at 60 days demonstrated complete urothelial coverage with a mean epithelial thickness of 45.2 ± 8.7 μm, an inflammation score of 1.25 ± 0.5, and a fibrotic area percentage of 18.4 ± 5.2% (Table 3).

When benchmarked against literature values for small intestinal submucosa (SIS) scaffolds, the 3D-printed construct achieved faster epithelialization (60 days vs. 90 days), exhibited comparable inflammation profiles, and was associated with no mortality compared to 15%–20% reported for SIS (Table 4). Surgical outcomes were uniformly favorable, with no leakage events (0/4 animals), full suture retention, and absence of tension signs across all cases (Table 5).

### 3.2. Postoperative Recovery and Functional Outcomes

All experimental animals exhibited uncomplicated postoperative recovery, maintaining 97.8 ± 2.1% of preoperative body weight at the 60-day endpoint with normal activity and feeding behaviors. The regenerated urothelium displayed histoarchitectural features—including uniform stratification and the absence of ulceration—that correlated with functional barrier formation, as evidenced by zero cases of urinary leakage or hydronephrosis. However, definitive assessment of barrier competency (e.g., via uroplakin immunohistochemistry or transepithelial resistance measurements) remains a target for future investigation.

### 3.3. Comparative Context

While the study did not employ direct contemporaneous controls, experimental outcomes were rigorously benchmarked against published standards for rabbit bladder repair models. Key metrics—such as epithelialization timelines (60 days vs. 90 days in SIS grafts) and inflammation profiles—were contextualized within the broader literature on bladder tissue engineering (see Table 6 for comparative analysis).

## 4. Discussion

Cystoplasty remains essential for managing bladder dysfunction; however, traditional enterocystoplasty carries significant risks, including metabolic complications, stone formation, and mucous production [9–11, 14, 15]. Tissue engineering approaches—particularly 3D bioprinting—offer promising alternatives by enabling patient-specific scaffolds with precise anatomical customization [22–24, 34, 35]. In this study, we demonstrate the feasibility of low-cost 3D-printed multilayer scaffolds (PLA/TPU/PVA) for bladder reconstruction, leveraging their tunable degradation profiles and mechanical properties [26–29]. Notably, all animals achieved complete reepithelialization within 60 days without urinary leakage, an outcome that compares favorably to biologically derived matrices (BAM/SIS), where leakage and mortality rates can approach 20% [6, 9–11].

While these results are encouraging, several limitations must be considered. The limited sample size (*n* = 4) restricts statistical generalizability, and the absence of direct contemporaneous controls necessitates reliance on historical benchmarks. Nonetheless, outcomes were rigorously contextualized against established bladder repair models, and the use of FDA-approved materials (PLA/TPU) provided validated baselines. Universal cystolithiasis (4/4 animals) likely arose from the interfacial microarchitecture (Figure 2(b)), species-specific alkaline urine (rabbit pH ∼8.5), and degradation byproduct release. Micro-CT confirmed calcium phosphate composition, while sterile processes were inferred from the absence of clinical infection, chronic inflammation patterns, and homogeneous stone microstructure. This complication is consistent with rabbit model rates for biological scaffolds (75%–100% [30]), suggesting a strong model-specific contribution.

The study's focus on structural endpoints omitted urodynamic evaluations (capacity and compliance) and barrier functionality verification (e.g., uroplakin/ZO-1 staining). Nevertheless, the observation of uniform epithelial stratification (45.2 ± 8.7 μm) and zero leakage implied functional barrier formation. Mechanical testing established tensile properties under dry conditions (modulus: 1.2 GPa vs. native bladder: ∼0.25 MPa [49]) but did not include physiological metrics such as burst pressure or suture retention. Degradation was inferred indirectly from a 12% strength reduction at 60 days, without layer-specific or temporal resolution.

Future research will focus on optimizing material design by refining surface coatings and porosity to mitigate lithiasis and integrating bioactive molecules to enhance regeneration [19, 21, 28]. Advanced characterization will involve layer-specific degradation kinetics in ISO-simulated urine, μCT/SEM microstructural analysis, and hydrated mechanical testing for burst pressure and cyclic compliance. Functional validation will include uroplakin and α-SMA immunohistochemistry, transepithelial resistance testing, urodynamic assessment, and metabolic cage studies. Translational models in pigs (pH ∼6.5) will help differentiate material-specific from species-specific effects, with larger cohorts and longitudinal designs (6–12 months) to monitor anastomotic integrity [50].

In conclusion, this cost-effective bioprinting platform advances the application of synthetic scaffolds in complex reconstructive scenarios such as bladder exstrophy [3, 4]. Although stone formation and limited functional data temper immediate clinical extrapolation, the absence of leakage, rapid epithelialization, and mild inflammation (mean score: 1.25; α-SMA+ in 75% of specimens) highlight its translational potential. Future iterations incorporating autologous cells or dynamic mechanical conditioning may bridge the gap between structural repair and functional restoration, ultimately providing personalized alternatives to enterocystoplasty.

## 5. Conclusion

This study establishes the feasibility, preliminary safety, and translational potential of low-cost 3D-printed PLA/TPU/PVA scaffolds for bladder augmentation in rabbits, demonstrating no urinary leakage, minimal adhesions, and complete reepithelialization within 60 days. Compared with biological matrices (BAM/SIS), these synthetic scaffolds achieved similar inflammatory profiles, lower mortality rates, and favorable integration, albeit with a modestly higher incidence of cystolithiasis. The constructs exhibited promising biocompatibility, mechanical durability, and anatomical conformity, supporting their suitability for complex urologic reconstruction such as bladder exstrophy. Nonetheless, the small sample size, short follow-up duration, and absence of functional assessments (e.g., urodynamics) limit definitive conclusions. Addressing these gaps—particularly through larger cohorts, longer-term studies, and targeted strategies to mitigate stone formation—will be essential to fully validate these scaffolds as a clinically viable alternative to traditional enterocystoplasty.

## Figures and Tables

**Table 1:** Comparison of mechanical properties between used and unused (test) bladder scaffolds following tensile testing.

	Force (N)		Extension (mm)		Stress (MPa)		Elongation (%)		Module (MPa)	
	Mean	Standard deviation	Mean	Standard deviation	Mean	Standard deviation	Mean	Standard deviation	Mean	Standard deviation
Used scaffold	68.6	10.4	4.727	0.591	2.80	0.496	25.84	4.2	12.362	1.78
Unused (test) scaffold	68.1	9.7	4.003	0.457	2.78	0.397	26.72	3.5	11.344	1.22

*Note:* Key parameters include force, extension, stress, elongation percentage, and modulus of elasticity.

**Table 2:** The scaffold dimensional specifications.

Parameter	Value
PLA base layer	0.5 mm
TPU intermediate layer	0.5 mm
PVA top layer	0.2 mm
Pore geometry	Hexagonal honeycomb pattern
Average pore size	150–200 μm

**Table 3:** Quantitative histomorphometry.

Parameter	Mean value ± STD
Epithelial thickness	45.2 ± 8.7 μm
Inflammation score	1.25 ± 0.5
Fibrotic area percentage	18.4 ± 5.2%

**Table 4:** Literature comparison (SIS vs. our scaffold).

Outcome	SIS	Our scaffold
Epithelialization time	90 days [30]	60 days
Inflammation	Comparable	Comparable
Mortality	15%–20% [33]	0%

**Table 5:** Surgical outcomes.

Parameter	Results
Leakage events	0/4
Suture retention	100%
Tension sign	Not observed

**Table 6:** Summary of histopathological and postoperative findings following cystoplasty using 3D-printed scaffolds in four rabbits.

Variables	No 1	No 2	No 3	No 4
Sex	Male	Female	Male	Female
Reepithelialization	Complete	Complete	Complete	Complete
Inflammation	Absent	Mild chronic and acute	Mild acute and chronic	Mild acute and chronic
Fibrosis	Mild	Moderate	Mild	Mild
Ulceration	Absent	Present	Present	Absent
Stone	Absent	Present	Present	Present

*Note:* Parameters include reepithelialization, inflammation, fibrosis, ulceration, and stone formation.

## Data Availability

The data that support the findings of this study are available from the corresponding author upon reasonable request.
